# Current Knowledge on Insecticide Resistance in *Aedes albopictus* (Skuse, 1894) in Latin America and the Caribbean Nations

**DOI:** 10.3390/insects17030264

**Published:** 2026-03-01

**Authors:** João Simão Silva Gonçalves, Ademir J. Martins, Vincent Corbel, Laura Harburguer, Christian R. Gonzalez, Cynara Melo Rodovalho, José Bento Pereira Lima

**Affiliations:** 1 Laboratório de Biologia, Controle e Vigilância de Insetos Vetores, Instituto Oswaldo Cruz, Fundação Oswaldo Cruz-Fiocruz, Rio de Janeiro 21040-360, Brazil; 2 Maladies Infectieuses et Vecteurs: Écologie, Génétique, Évolution et Contrôle, Institut de Recherche pour le Développement, Centre National de la Recherche Scientifique, Université de Montpellier, 34394 Montpellier, France; 3 Centro de Investigaciones de Plagas e Insecticidas (CIPEIN-UNIDEF/CITEDEF/CONICET), Villa Martelli, Buenos Aires 1603, Argentina; 4 Instituto de Entomología, Facultad de Ciencias Básicas, Universidad Metropolitana de Ciencias de la Educación, Ñuñoa, Santiago 7760197, Chile

**Keywords:** insecticide susceptibility, surveillance, review, vector control

## Abstract

*Aedes albopictus* is an important vector of several arboviruses and has expanded its distribution throughout Latin America and the Caribbean in recent decades. Despite its increasing epidemiological relevance, data on insecticide resistance in this region remain limited compared to *Aedes aegypti*. This review compiled and analyzed the published evidence on this topic across the region. A literature search was conducted in PubMed, SciELO, Google Scholar, LILACS, and DOAJ, using combinations of the terms “insecticide resistance” or “insecticide susceptibility” plus “*Aedes albopictus*”, and the names of all countries recognized by the United Nations as part of the region. Studies published until May 2025 were considered, regardless of the publication year. Following the core PRISMA principles adapted for this review, 33 studies were identified, of which 17 met the inclusion criteria. Results indicate recurrent but uneven reports of resistance, with organophosphates being the most frequently evaluated insecticide (33.9%), followed by pyrethroids (27.7%) and carbamates (10.8%), and metabolic mechanisms being the most implicated. Evidence suggests that control actions focused on *Ae. aegypti* may also impose selective pressure over *Ae. albopictus*. Overall, the resistance found on *Ae. albopictus* emphasizes the need for expanded and standardized surveillance to support effective vector control across the Americas.

## Simple Summary

The mosquito *Aedes albopictus*, also known as the Asian tiger mosquito, has become increasingly common in many countries in Latin America and the Caribbean and can be the vector of several pathogens, such as dengue, chikungunya, and Zika viruses. As health authorities often rely on chemical insecticides to control mosquitoes, little is known about how this mosquito responds to these products in this region. In this review, we gathered and examined studies from multiple countries to provide an overview of the current knowledge on insecticide susceptibility in this species. The results are unevenly distributed and suggest that some mosquito populations are starting to survive treatments that were once fully effective, with most information coming from Brazil and Mexico, while large parts of the region remain poorly studied. This lack of information may hinder effective mosquito control and highlights the need for broader and more systematic monitoring efforts so that control actions can be better planned and adapted, helping protect communities from mosquito-borne diseases.

## 1. Introduction

The Asian tiger mosquito, *Aedes (Stegomyia) albopictus* (Skuse, 1894), is an invasive species widely recognized for its medical importance due to its ability to transmit arboviruses, including dengue, chikungunya, and Zika [1,2]. Although often considered a secondary vector of arboviruses in tropical endemic regions where *Aedes aegypti* is abundant, *Ae. albopictus* plays a primary epidemiological role in temperate regions, like Asia and Europe [3].

Originating from Southeast Asia, *Aedes albopictus* has become one of the most successful invasive mosquito species worldwide [4]. Its global spread, now encompassing all continents except Antarctica, has been largely driven by international trade, particularly the transport of used tires and ornamental plants that facilitate the passive movement of eggs and larvae. This rapid expansion has been sustained by the species’ remarkable ecological plasticity and outstanding ecological adaptability, allowing it to survive in both tropical and temperate zones, due to its adaptation to colder climates and urban, peri-urban, and rural habitats [5,6]. Its impressive capacity for egg diapause over winter contributes to the establishment of stable populations and, in some regions, to the autochthonous transmission of arboviruses [7,8].

This ecological flexibility is particularly relevant for the colonization of vast areas of South America (e.g., Chile, Peru, and southern areas of Argentina), where winters have low average temperatures [9]. Similar patterns have been observed elsewhere in the world, given that *Ae. albopictus* has been responsible for outbreaks of dengue and chikungunya, as recently observed in Southern Europe [10]. According to the European Centre for Disease Prevention and Control (ECDC), *Ae. albopictus* is currently established in 16 European countries [11].

Although traditionally associated with natural habitats for feeding and oviposition, for a long time it was considered a rural vector, but its presence has increasingly been recorded in urban areas, with larvae developing in artificial containers in sympatry with *Ae. aegypti* [12]. Urbanized environments provide a greater variety of containers for larval habitats and more blood sources for adult mosquitoes. Where both species share breeding sites, interspecific competition may occur, influencing local mosquito population dynamics [13,14,15,16,17].

On a broader scale, global drivers, such as climate change and rapid urban growth, have substantially contributed to the geographical expansion and permanent establishment of *Ae*. *albopictus* in new regions of the world. Rising average temperatures and reduced seasonality favour winter survival, accelerate the life cycle, and extend the window for arbovirus transmission, even in areas previously considered climatically unsuitable for the species. Concurrently, unplanned urbanization increases the availability of artificial breeding sites and encourages reliance on chemical interventions for vector control [18,19].

In the Americas, *Ae. albopictus* was first recorded in the United States in 1983 [20]. Soon after, the species spread throughout the continent, with the earliest records in Latin America reported in Brazil (1986) and Mexico (1988) [21,22]. During the 1990s, its presence was documented in several additional countries, including Argentina, Colombia, Cuba, the Dominican Republic, El Salvador, Guatemala, Honduras, and Paraguay. This expansion continued through the 2000s and 2010s with new records in Belize, Costa Rica, Ecuador, Haiti, Jamaica, Nicaragua, Panama, Trinidad and Tobago, Uruguay, and Venezuela [23,24]. Most recently, it was confirmed in Bolivia in 2023, underscoring the ongoing nature of its continental expansion [25].

Although the geographic expansion of *Ae. albopictus* in Latin America and in the Caribbean States is well documented, studies on its biology and resistance to insecticides are still limited. This gap is due to the historical classification of the mosquito as a secondary vector in tropical and subtropical regions, where surveillance and control programs usually focus on *Ae. aegypti* [24,26].

Most arbovirus control programs focus on controlling the vector, which can be achieved in several ways, with chemical control being the most widespread method worldwide. This method uses compounds of organic or inorganic origin and, when used correctly in sensitive populations, can end outbreaks of diseases transmitted by these vectors. However, its intensive use leads to great pressure and the selection of resistant insect populations [27,28].

Given the widespread use of insecticides, it is important to present the main classes currently used against *Aedes* mosquitoes [29] and their modes of action. Organochlorides and pyrethroids target voltage-gated sodium channels, keeping them open and hereby inducing repetitive nerve impulses that lead to paralysis and death [30,31]. Carbamates and organophosphates inhibit the enzyme acetylcholinesterase (AChE) at the synapse, blocking nerve signal impulses [32]. Neonicotinoids and butenolides activate postsynaptic nicotinic acetylcholine receptors (nAChRs), resulting in continuous nerve stimulation, paralysis, and death [33,34]. Pyrroles disrupt oxidative phosphorylation in mitochondria, thereby impairing ATP production. The resulting loss of energy causes cellular dysfunction and death in mosquitoes [35].

Among the larvicides there are: the spinosyns, which are derived from the actinobacterium *Sacharopolyspora spinosa,* and act by disrupting the nicotinic acetylcholine receptors (nAChRs), affecting neural transmission [36]; the products based on *Bacillus thuringiensis* var. *israelensis* (*Bti*), that target the insect’s midgut, binding to epithelial cells and disrupting their osmotic balance, which leads to cell lysis and death [37]; and the insect growth regulators (IGRs), which can be classified in two main types, the juvenile hormone analogues (JHA) that block metamorphosis, preventing the emergence of reproductive adults, and chitin synthesis inhibitors (CSI) which disrupt the formation of the exoskeleton, causing the death of the insect [38,39].

Tracking the emergence and spread of insecticide resistance in arthropod vectors is essential in order to recommend the most appropriate insecticides for national control programmes [40]. Insecticide resistance is a heritable trait that enables mosquitoes to survive exposure to an insecticide that would normally kill them [29]. It can result from multiple mechanisms, including behavioural adaptations and physiological changes, such as overexpression of detoxifying enzymes, target-site mutations (single nucleotide polymorphisms in the target protein gene) and changes in the chemical composition of the insect’s cuticle (reduced penetration of insecticides) [41,42,43,44]. It can be classified into levels, and these levels are commonly quantified using resistance ratios (RRs), calculated as the ratio between lethal concentrations obtained for field populations and those observed in a susceptible reference strain [45].

Most chemical control strategies in the American continent are primarily designed to target *Ae. aegypti*, but *Ae. albopictus* frequently share the same breeding sites and adult habitats. As a result, this species may be indirectly exposed to insecticides, and the survival of resistant individuals may be favored through bystander selection, a phenomenon in which resistance becomes evident in a non-target species due to exposure to control methods aimed at another species [24,26,46]. In addition, the extensive and prolonged use of chemical compounds in public health campaigns and agricultural practices increases the intensity and continuity of selective pressure across urban, peri-urban, and rural environments [47,48].

Due to *Ae. albopictus’* geographical expansion and the increasing overlap of control interventions targeting multiple mosquito species within the same environments, we aim to summarize the current knowledge on insecticide resistance of *Ae. albopictus* in Latin America and Caribbean nations. Although a recent review examined the resistance status of this species in North America, no equivalent synthesis is available for Latin American and Caribbean countries [49]. To our knowledge, this review provides the most up-to-date overview of the insecticide resistance of this mosquito in the above-mentioned region.

## 2. Materials and Methods

### 2.1. Bibliographical Research and Criteria for Data Inclusion in the Survey

Data were extracted from the online databases PubMed, Google Scholar, Scielo, Lilacs, and Doaj. Searches were conducted between March and May 2025 and included all articles published up to May 2025, with no restrictions on the year of publication due to the scarcity of data on the theme. The terms used in the search were “insecticide resistance *Aedes albopictus*” and “insecticide susceptibility *Aedes albopictus*”, combined with the name of each Latin American and Caribbean nation as listed by to The United Nations website: Antigua and Barbuda, Argentina, Bahamas, Barbados, Belize, Bolivia, Brazil, Chile, Colombia, Costa Rica, Cuba, Dominica, Dominican Republic, Ecuador, El Salvador, Grenada, Guatemala, Guyana, Haiti, Honduras, Jamaica, Mexico, Nicaragua, Panama, Paraguay, Peru, Saint Kitts and Nevis, Saint Lucia, Saint Vincent and the Grenadines, Suriname, Trinidad and Tobago, Uruguay and Venezuela, according [50].

Studies were eligible for inclusion if they reported empirical data derived from biological, biochemical, or molecular assays conducted directly. Studies that relied solely on theoretical assessments, modelling approaches, or indirect data were not included in our results, because they were not an evaluation of the species’ susceptibility profile to the tested compounds, which can reduce the applicability of the findings in a real-world vector control scenario. Studies reporting control mortality above recommended thresholds were excluded because of the reduced quality of the resulting evidence.

We chose to restrict our analysis to data published in scientific journals in order to ensure transparency, traceability, and prior peer review. This decision is particularly important because, although some countries routinely perform insecticide susceptibility assays, the resulting data are not always made publicly available, which could bias the current understanding of resistance across the continent. In addition, access to institutional reports may depend on specific agreements that fall beyond the scope of this study, and documents that are not published in the academic literature may not have undergone peer review. Thus, by prioritizing evidence reported in peer-reviewed scientific publications, we aim to provide more robust and comparable conclusions.

### 2.2. Methodological Heterogeneity Assessment and Data Interpretation

Until 2022, no WHO-standardized test protocol or recommended discriminating concentrations were available for *Ae. albopictus* [29], which makes resistance studies on this species particularly challenging. Consequently, a certain level of methodological variability cannot be excluded from the bioassay conditions, diagnostic endpoints, and resistance criteria. Studies that lacked a susceptible reference strain, and/or used synergists, were included because they provided empirical data on *Ae. albopictus* susceptibility or resistance to insecticides. In such cases, resistance or susceptibility was recorded and summarized according to the criteria and interpretations reported in the original publications.

When available, data on control mortality, application of Abbott’s correction, resistance/susceptibility metrics (e.g., resistance ratios), information on mosquito collection methods (e.g., larvae, ovitraps, or adult collections), and units of insecticide exposure (e.g., µg/bottle, mg/L, mortality percentage, or time to knockdown) were extracted to support interpretation. Because the diagnostic % mortality thresholds and reporting standards have changed over time, results were interpreted according to the criteria applied at the time of each study. This temporal variability was considered when synthesizing resistance patterns, considering the limitations of direct comparisons among studies conducted under different diagnostic frameworks.

A formal risk-of-bias or quality scoring tool was not applied because standardized protocols for monitoring insecticide resistance in *Ae. albopictus* across the Americas have only recently been established. Instead, study quality was assessed qualitatively during data extraction using the methodological indicators reported by the authors. Limitations related to study design were acknowledged when presented.

#### Interpretation of Biochemical and Molecular Resistance Markers

Biochemical and molecular data were interpreted according to the criteria and conclusions presented by the original authors, supported by established associations with insecticide resistance mechanisms in *Aedes* mosquitoes (Table 1). Elevated activity of detoxification enzymes, reflecting enzyme-mediated degradation or sequestration of insecticide particles prior to target-site interaction, was interpreted as indicative of metabolic resistance. Overexpression of mixed-function oxidases (MFOs), particularly cytochrome P450 monooxygenases (P450s), esterases (α-EST and β-EST), and glutathione S-transferases (GSTs) has been widely associated with resistance to pyrethroids, organophosphates, and carbamates and was interpreted accordingly [51,52,53].

Alterations in acetylcholinesterase (AChE) are implicated in insecticide resistance through both *metabolic* mechanisms related to enzyme inhibition as well as molecular mechanisms, involving gene mutations that alter its structure and function, and are commonly associated with resistance to organophosphate and carbamate insecticides. Molecular analyses typically focus on mutations in genes encoding insecticide target sites that reduce binding affinity and efficacy. The most frequently investigated mutations occur in the voltage-gated sodium channel gene (Nav) at positions previously implicated in knockdown resistance (kdr), which is primarily associated with resistance to pyrethroids and organochlorides [51,53,54].

## 3. Results

### 3.1. Study Selection

Abstracts were screened, and full-text copies were retrieved for all studies considered potentially relevant. From each eligible study, we extracted information on the insecticides evaluated in bioassays, the enzymes assessed in biochemical assays, the genetic targets analysed in molecular assays, the field populations tested, the presence or absence of susceptible reference strains, and details of field collections.

It is worth highlighting that many studies have described in their methodology not only assays with *Ae. albopictus* but also assays with *Ae. aegypti* and/or other vectors. However, since the objective of this study was specifically to address the resistance of *Ae. albopictus* to public health insecticides, only the results for this species are presented.

**Figure 1:**
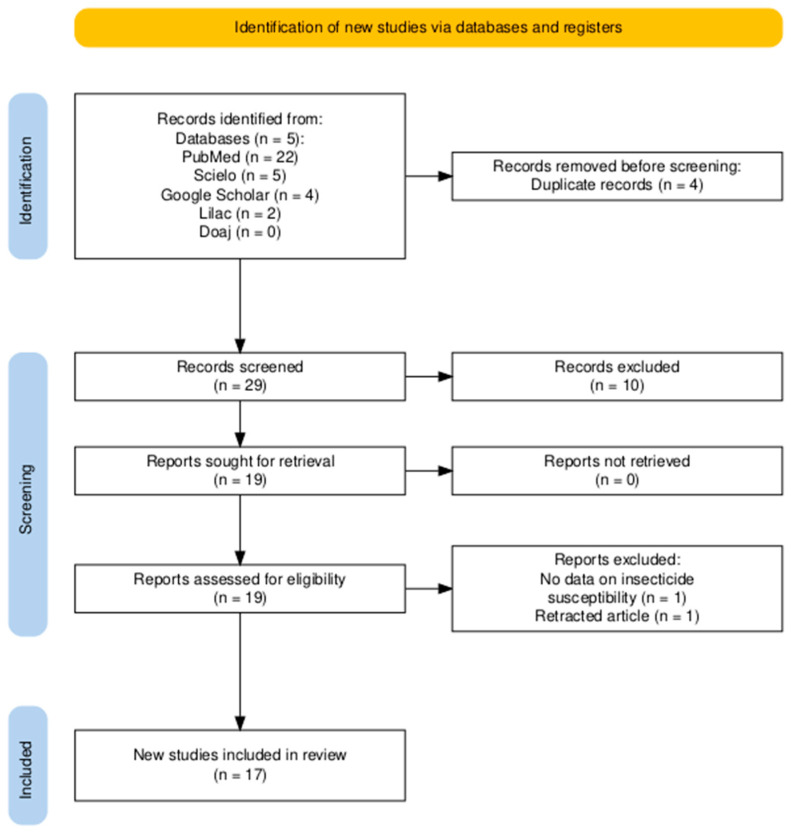
PRISMA flow chart summarizing the study selection process conducted in June 2025 for the review on *Aedes albopictus* insecticide resistance in Latin America and the Caribbean nations. No automation tools were used [55].

The electronic search initially identified 33 articles. After removing duplicates and excluding studies that did not provide evidence of *Ae. albopictus* insecticide susceptibility or resistance in Latin American and Caribbean countries, 17 studies remained for full review (Figure 1).

### 3.2. Studies’ Overview

**Figure 2:**
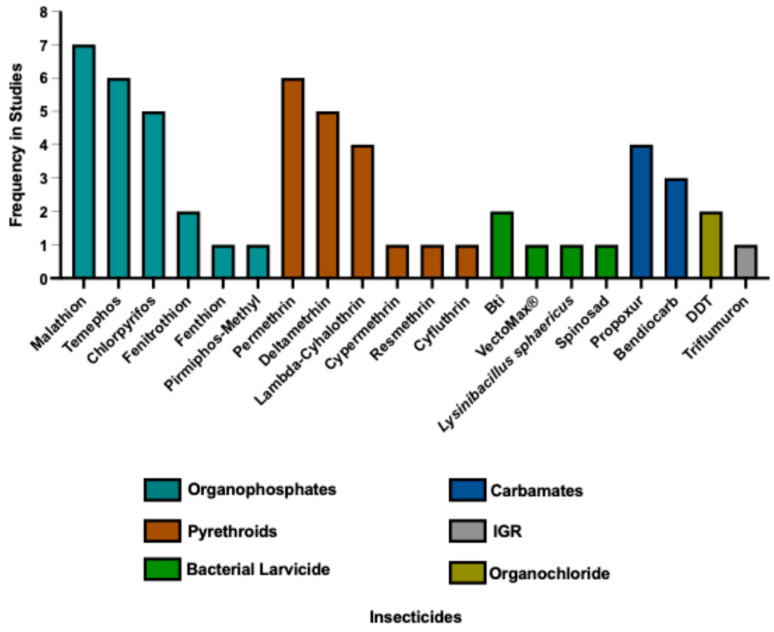
Distribution of insecticides evaluated in susceptibility and resistance assays with *Aedes albopictus* in Latin America and the Caribbean. Data are derived from 17 studies, encompassing 65 mosquito populations (including susceptible reference strains), published between 1990 and 2025. The figure summarizes the frequency with which each insecticide was tested across the included studies.

Across Latin America, studies on *Ae. albopictus* insecticide resistance reveals a heterogeneous landscape influenced by geography, historical insecticide use, and local coexistence with *Ae. aegypti*. No study reported control mortality above recommended thresholds. Overall, organophosphates, mainly malathion and temephos, were the most frequently tested compounds, followed by pyrethroids and carbamates. All insecticides evaluated in the studies included in this review are shown in Figure 2. The frequency of the insecticides does not necessarily reflect their use in vector control programs.

**Figure 3:**
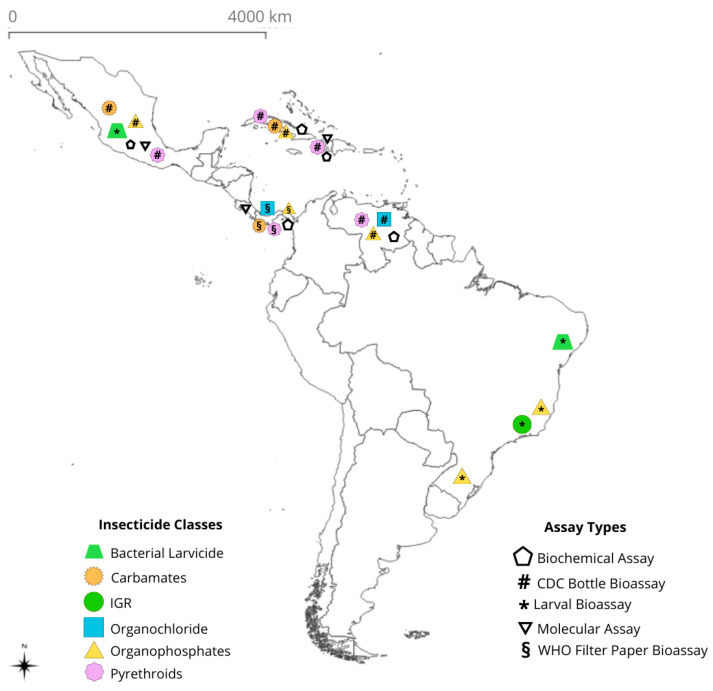
Insecticide classes (**left**) and assay types (**right**) used in studies evaluating insecticide resistance in *Aedes albopictus* in Latin America and the Caribbean. Data are derived from 17 studies, encompassing 65 mosquito populations (including susceptible reference strains), published between 1990 and 2025.

Figure 3 presents the class of insecticides tested sorted by country, providing an overview of the heterogeneity in chemical control strategies across the region. In addition, the map indicates the countries where molecular and/or biochemical assays were conducted. This combined visualization highlights the influence of local epidemiological context and the historical reliance on specific compounds, allowing a better understanding regarding the geographic scope of resistance studies.

Among the studies reviewed, the CDC bottle bioassay emerged as the most employed method for evaluating insecticide resistance in *Ae. albopictus*, which is widely used due to its simplicity, cost-effectiveness, and suitability for field and laboratory conditions, making it a valuable tool for resistance monitoring programs [56]. Differences in endpoints and discriminant concentrations across studies are acknowledged and considered in data interpretation (see Section 2.2).

Table 2 summarizes the key characteristics of the studies included in this review: insecticide class and active ingredients tested, their concentrations when disclosed in the original articles, the type of bioassay performed, and the main outcomes observed. Additional methodological details are provided in the Supplementary Material. 

### 3.3. Countries

**Figure 4:**
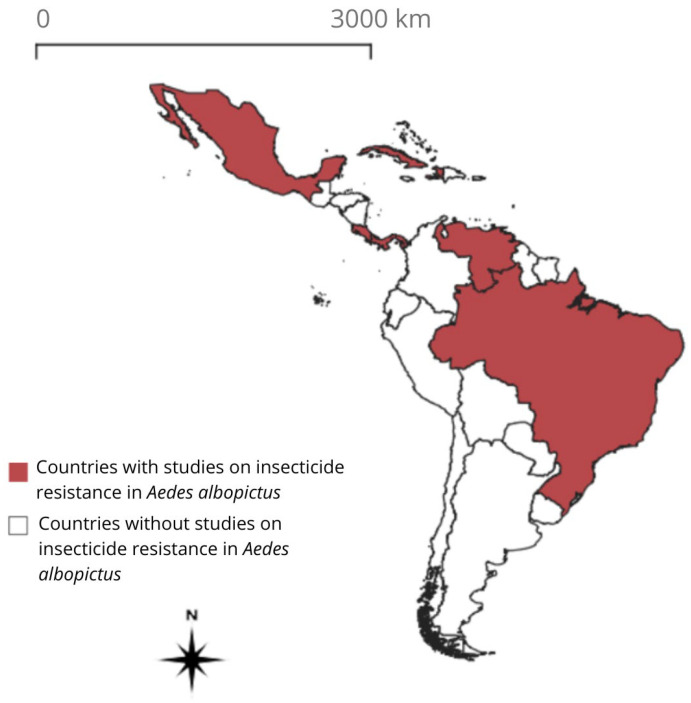
Countries with resistance data on *Aedes albopictus* across Latin America and the Caribbean nations (red).

From the 33 nations that are part of Latin America and the Caribbean [50], we found eight studies in Mexico, four studies in Brazil, one in Costa Rica, one in Cuba, one in Haiti, one in Panama, and one in Venezuela (Figure 4). The manuscripts were published between 1990 (Brazil) and 2024 (Mexico).

One study from Argentina was excluded because it did not assess the insecticide resistance in natural populations from the region. Instead, it compared the insecticide susceptibility of the Rockefeller strain derived from Venezuela with that of a susceptible *Ae. albopictus* strain from Gainesville, FL, USA, and therefore did not represent populations from Latin America [74].

#### 3.3.1. Brazil

The first report of insecticide resistance in *Ae. albopictus* in Brazil was published in 1990. Wesson performed dose response mortality tests following the WHO protocol from 1981 [75] with larvae from 25 field populations, including 13 populations from the United States, five from Brazil, two from Southeast Asia, and five from Japan. In addition, two laboratory lineages, ROCK from the USA and OAHU from Hawaii, were also tested against the organophosphates temephos (0.01 mg/L), chlorpyrifos (0.02 mg/L), fenitrothion (0.02 mg/L), fenthion (0.20 mg/L), and malathion (0.02 mg/L). Diagnostic doses were determined using a laboratory lineage. The value obtained was subsequently doubled, following the WHO recommendations in force at the time for *Ae. aegypti*. All populations showed LC_95_ values below the diagnostic concentrations for the tested insecticides, indicating susceptibility, except one from Anchieta, Espirito Santo, whose LC_95_ exceeded the diagnostic concentration for chlorpyrifos, indicating resistance as concluded by the authors [57].

Two decades later, in 2011, Prophiro et al. [58] investigated the coexistence of *Ae. aegypti* and *Ae. albopictus* populations in the municipalities of Ubiratã, Santa Helena and Foz do Iguaçu, all belonging to Paraná State, and the city of Tubarão, located in Santa Catarina State. They assessed larval susceptibility of the populations found in these locations to temephos and, due to the lack of a reference lineage of *Ae. albopictus* for comparison, the *Ae. aegypti*’s Rockefeller reference lineage was used. The diagnostic dose (0.006 mg/L) was previously calculated and corresponded to twice the CL_99_ of the Rockefeller susceptible strain. Based on the diagnostic concentration criteria, mortality above 98% indicating susceptibility, 80-98% indicating incipient resistance, and below 80% indicating resistance, all tested populations showed mortality between 87–96%, consistent with an incipient resistance status. Resistance levels were further classified as low, since all RR_95_ values were below 5.0. Interestingly, in Tubarão, there was no recorded history of temephos application up to that point, suggesting that resistance may have emerged through cross-selection or due to gene flow from neighboring populations.

In 2013, Belinato et al. [59] evaluated the efficacy of triflumuron against laboratory lineages of *Ae. albopictus* and *Culex quinquefasciatus* and field populations of *Ae. aegypti*. The *Ae. albopictus* lineage was collected from Rio de Janeiro and maintained in the laboratory for five years; however, it was not considered as an insecticide susceptible reference lineage. Thirteen concentrations that ranged from 0.25 μg/L to 4.5 μg/L were used in the dose–response bioassays. The results showed higher larval mortality rates at the highest triflumuron doses, from 3.5 μg/L to 4.5 μg/L. In the dosages between 1.25 μg/L and 3.5 μg/L, pupal mortality was evident, while doses up to 3.0 μg/L resulted in increased adult mortality. In conclusion, the insecticide effectively inhibited adult emergence in a dose-dependent manner under laboratory conditions, with effective emergence inhibition concentrations of 1.59 µg/L (EI_50_) and 2.63 µg/L (EI_90_). The lack of standardized discriminant concentrations for triflumuron or comparable reference values limits the interpretation of whether these values indicate susceptibility.

The most recent study found was published in 2017 by Suter et al. [60]. The authors assessed third-instar larvae from a field population (SPLab) originated from Sítio dos Pintos, Recife, and a lab lineage (RecLab) established at the Instituto Aggeu Magalhães from eggs collected in Recife. The larvae were exposed to *Bti* (aqueous suspensions at 5 g/L), *Lysinibacillus sphaericus*, the bioinsecticide *VectoMax* CG^®^ (a formulation that combines *Bti*’s advantage of resistance-blocking together with *L. sphaericus* advantage of longer residuality) and to the isolated toxins Cry11Aa and Cry4Ba. All populations were classified as susceptible to *Bti* and its isolated toxins, once RR values stayed consistently below two and LC_50_ estimates for the isolated toxins were similar to those found on the reference colony. The bacterium *L. sphaericus*, which is well documented for *Ae. aegypti* and poorly documented for *Ae. albopictus* control, also proved to be effective. Finally, the authors concluded that the combination product *VectoMax* CG^®^ proved to be a promising option for vector control.

#### 3.3.2. Costa Rica

In Costa Rica, the only study found was from Chaves et al. (2015) [61], who collected mosquitoes from Sarapiquí, the only location in the country where a persistent infestation of the species had been identified up to date. A total of 58 mosquitoes were collected, and DNA was successfully extracted from 56 of them. No kdr mutation, which is commonly related to pyrethroid resistance, was detected.

#### 3.3.3. Cuba

In 2023, Piedra et al. [62] published the results of adult bioassays, using the CDC bottle bioassay (2010), with the doses being previously established in their laboratory, against deltamethrin (6.5 μg/bottle), cypermethrin (13.5 μg/bottle), lambda-cyhalothrin (6.5 μg/bottle), chlorpyrifos (90 μg/bottle), propoxur (15 μg/bottle) and bendiocarb (12 μg/bottle), as well as larval bioassays for temephos (0.08–0.116 ppm) susceptibility, conducted on two *Ae. albopictus* populations (Plaza and Boyeros) collected in Havana, Cuba, in 2019. The Fraga lineage, maintained at the Institute of Tropical Medicine Pedro Kouri (IPK) since 2012, was used as a susceptible reference. The resistance ratio (RR_50_) was calculated comparing the value of the LC_50_ of the field colonies with the Fraga strain. Based on the RR_50_, both field populations showed high resistance to temephos and incipient resistance to deltamethrin (mortality between 90% and 95% in the bottle bioassays), while the Plaza population also exhibited resistance to lambda-cyhalothrin (90% mortality in the test). All populations remained susceptible to cypermethrin, chlorpyrifos, propoxur and bendiocarb.

Biochemical assays were also performed and demonstrated an increased activity of α-esterase (α-EST) and glutathione S-transferase (GST) enzymes in resistant populations when compared with the susceptible reference strain, thus associated with temephos resistance. In the study, the authors further note that this enzymatic profile is consistent with previous reports in studies from Asia and Europe. Resistance to pyrethroids was likely influenced by their widespread use in the control of *Ae. aegypti*, potentially promoting resistance in *Ae. albopictus* in environments where both species coexist.

#### 3.3.4. Haiti

In 2012, the first, and so far, only report of pyrethroid resistance in *Ae. albopictus* in Haiti was published. In 2010, McAllister et al. [63] collected mosquito larvae and eggs from two sites in Port-au-Prince: one in the U.S. embassy (site A) and one in an adjacent neighbourhood (site B). Due to the absence of insecticide treatment in the Port-au-Prince area, three common insecticides used in vector control programs elsewhere were selected for testing: the pyrethroids permethrin and deltamethrin, and the organophosphate malathion.

CDC bottle bioassays using the Brogdon and McAllister methodology were conducted using CDC doses of 15 μg permethrin, 10 μg deltamethrin, or μg of malathion, each dissolved in acetone. Assays were performed using a mixed group of adult mosquitoes, composed of 66% *Ae. aegypti* and 34% *Ae. albopictus*. Since adult mosquitoes reared from eggs and larvae collected at both sites A and B were combined for the initial bioassays, it is not possible to determine the specific origin of the individuals within the mixed group. However, it is known that only *Ae. aegypti* emerged from Site A, and only this site was tested against all three insecticides. Thus, *Ae. albopictus* was tested only against permethrin.

When exposed to the pyrethroid, 15% of the adults from the mixed population (12/78) survived the exposure beyond the resistance threshold time of 30 min. Biochemical assays were therefore conducted exclusively on females, in accordance with WHO recommendations. Although the initial study design aimed to include both male and female *Ae. albopictus* to enable comparison between both sexes of *Ae. aegypti*, this was not feasible due to the insufficient number of male *Ae. albopictus* obtained from field collections. The results showed elevated oxidase levels in males *Ae. albopictus* when compared to male and female *Ae. aegypti*, as well as slightly higher levels of α-EST, GST and acetylcholinesterase (AChE) compared to *Ae. aegypti* females, though similar to those observed in males. Finally, molecular assays did not detect the kdr mutations Val1016 Ile and Val1016Gly, but the V1011Met was found under high frequencies in *Ae. albopictus*. Overall, no resistance was detected in this study.

#### 3.3.5. Mexico

In 1996, Sames et al. [64] conducted one of the first bioassays involving *Ae. albopictus* in Latin America. Field populations were collected from three sites in Matamoros and one site in Reynosa, Mexico. For comparison, the susceptible lineage TAMU, from Texas A&M University, was used as the reference. The tested insecticides were malathion (concentrations ranged from 0.03 μg/L to 1.0 μg/L), chlorpyrifos (from 0.06 μg/L to 0.3 μg/L), resmethrin, and permethrin (from 0.06 μg/L to 0.3 μg/L for both) using bottle bioassays. The doses were determined using the insecticide-coated vial technique of Plapp and resistance ratios, RR_50_ and RR_95_, were calculated by dividing the field-strain LC_50_ or LC_95_ by the respective susceptible-strain LC_50_ or LC_95_. Based on the author’s experience and previous laboratory observations, RR values close to 10 were interpreted as indicative of incipient resistance, and all field-collected populations were found to be susceptible to the evaluated insecticides, based on their RR_50_ and RR_95_.

Almost 15 years later, in 2010, Marina et al. [65] tested spinosad, temephos and *Bti* against larvae of field populations of *Ae. aegypti* and *Ae. albopictus*. Although this study did not include laboratory-based biological assays, it provided important insights through field experiments evaluating the efficacy of these larvicides. The authors conducted field trials using black plastic containers filled with dechlorinated water placed in two cemeteries in southern Mexico, during the wet and dry seasons. Each container was treated with one of the following: spinosad (1 mg/L or 5 mg/L), temephos granules (0.1 g), *Bti* suspension (13 μL), or left untreated as controls. The experiments were conducted for 13 weeks per season in 2006, and the containers were arranged in specific Latin square designs. Weekly inspections recorded the number of live larvae and pupae, which were removed and identified after adult emergence. Eggs were also collected weekly and reared for species identification. Environmental variables such as water, air temperature, and humidity were recorded at each sampling point. The results demonstrated that spinosad (suspension concentrate) and temephos (granular sustained-release formulation) were both effective in preventing the development of *Ae. albopictus* in both seasons. In contrast, the *Bti* suspension lost activity over time [65].

In 2020, two studies explored insecticide resistance in *Ae. albopictus* populations from Chiapas, Mexico. López-Solís et al. [66] conducted bioassays with mosquitoes collected from two urban sites in the city of Tapachula. Larvae were tested against temephos (0.012 mg/L) following the WHO protocol, whereas adult mosquitoes were evaluated using the CDC bottle bioassay protocol (2010). Diagnostic doses for bendiocarb (12.5 µg/bottle), permethrin (15 µg/bottle), malathion (50 µg/bottle), and deltamethrin (10 µg/bottle) followed CDC recommendations. Diagnostic doses for chlorpyrifos (60 µg/bottle) and propoxur (10 µg/bottle) were established by the laboratory. Biochemical assays assessed esterase, monooxygenase and GST levels, as well as the activity of AChE. All tests used a susceptible lineage from New Orleans (USA) as a reference. Populations were classified as resistant when mortality was <80%, as showing incipient resistance when ranged from 80–97%, and as susceptible when >98%, according to WHO criteria. Based on the mortality after 24 h exposure, both populations showed susceptibility to all carbamates evaluated, incipient resistance to permethrin, deltamethrin and chlorpyrifos, and resistance to malathion and temephos. According to the authors, the resistance to temephos may have occurred because it has been used for 30 years in the country against *Ae. aegypti*. Biochemical data corroborated these findings, as high levels of esterases and monooxygenases were observed in both populations. A reduction in acetylcholinesterase (AChE) activity was detected in both field populations based on biochemical assays. However, no phenotypic resistance to the tested carbamates was observed.

Tancredi et al. [67] traced the temporal and geographical distribution of resistance to pyrethroids from 512 *Ae. albopictus* specimens collected worldwide between 2011 and 2018. Of these, 182 were collected in Chiapas in 2016, and therefore, only those results will be shown in the present study. They searched for kdr mutations in the sodium channel gene and, as a result for the Mexican site, they detected mutations at positions 410, 989 and 1534 of the gene. Most individuals were heterozygotes, with a few carrying the double mutation S989Y-F1534L. Variants such as V410L and S989P were detected in heterozygosity at low frequencies (0.57%). These analyses of the mosquitoes from Chiapas, where the species was first reported in 2002, showed that they possessed genetic variability comparable to global populations and unique private alleles, suggesting genetic distinctiveness. Lower F_ST_ values compared to other invasive populations and Bayesian clustering revealed genetic admixture, with Chiapas populations presenting a low frequency of pyrethroid resistance-associated genotypes and being genetically similar to populations from Virginia, Greece, and northern Italy, supporting the hypothesis of multiple introduction events. The authors acknowledged the importance of conducting resistance bioassays to confirm any association between the alleles and phenotypic resistance but were unable to perform them due to logistical constraints.

In 2021, two other research papers were published. Janich et al. [68] investigated permethrin resistance using the CDC bottle bioassay protocol from 2013. Using concentrations that ranged from 0.5 to 5.0 μg/bottle, the authors estimated the lethal concentration (LC_50_) in eleven sites of Chiapas, including four in Tapachula, in a wide variety of containers associated with households (tires, cups, buckets, outdoor sinks) and seven in rural towns, including Puerto Madero, Huehuetán, Huixtla, Escuintla, Motozintla, Mapastepec and Pijijiapan, where the immatures (larvae and pupae) were collected in flowerpots and vases within cemeteries. The susceptible control lineage used was ATM-NJ95 obtained from the Biodefense and Emerging Infections Research Resources Repository (BEI Resources). Molecular assays screened for the F1534C kdr mutation in the voltage-gated sodium channel (vgsc) gene and biochemical assays were conducted with a permethrin-selected lineage, obtained from the survivors of the 11 populations exposed to the insecticide and repeatedly selected at F5, F7, F9 and F11, and a non-selected control, obtained from the survivors of the 11 populations exposed to the insecticide at F3 and no longer exposed. The authors considered RRs as high when it was >10-fold, as moderate when between 5- and 10-fold, and low when <5-fold levels. Overall, all mosquitoes presented RRs between 1.0 and 3.0, showing a low level of permethrin resistance, and no significant differences were observed between the sites from within the city of Tapachula and rural/suburban sites outside of the city. All tested mosquitoes were homozygous for the wild-type allele of F1534 in the Nav gene, including the permethrin-selected population and the susceptible lineage ATM-NJ95. Since other studies showed that *Ae. aegypti* was highly resistant to permethrin in Mexico, the authors expected that *Ae. albopictus* would be subjected to the same insecticide selection pressure. However, this was not the case. Biochemical assays revealed low levels of enzymatic activity typically linked to pyrethroid resistance, and they hypothesised that the peri-urban and peridomestic area preference of *Ae. albopictus* may limit its exposure to insecticide selection pressure.

Contreras-Perera et al. [69] performed susceptibility tests with insecticides used for vector control in Mérida, Yucatán, Mexico. Field collections of pupae, larvae and adults of the mosquito were obtained in 2019 from an area with a confirmed presence of *Ae. albopictus.* They employed the 2010 CDC bottle test methodology, including a susceptible field population from Atlanta, Georgia, USA (donated by Emory University). They used the diagnostic doses indicated in the CDC guidelines for bendiocarb (12.5 μg/bottle), propoxur (12.5 μg/bottle), chlorpyrifos (85 μg/bottle), malathion (50 μg/bottle), permethrin (15 μg/bottle), and deltamethrin (10 μg/bottle). Populations were classified as resistant or susceptible according to the WHO criteria and the results indicated full susceptibility to the compounds evaluated, once mortality after 24 h of exposure was 100%.

Ciau-Mendonza et al. [70] published in 2022 a study evaluating the susceptibility status to temephos (0.012 mg/L) for *Ae. albopictus* larvae from four communities of Quintana Roo, Mexico using the WHO protocol and discriminant concentration for *Aedes* spp. Among the four populations tested (Cancún, Chetumal, Playa del Carmen and José María Morelos), Cancún and José María Morelos exhibited tolerance to the larvicide, while the other two remained susceptible. The authors applied the WHO criteria to confirm phenotypic resistance (mortality below 98% after 24 h of exposure to the insecticide). For comparison, they also performed the same bioassays with *Ae. aegypti* larvae from the same locations, which were all found to be resistant to the insecticide. Based on these findings, the authors concluded that *Ae. albopictus* had been exposed to the larvicide, though likely at a lower intensity than the other mosquito species.

The most recent Mexican paper in the field was published in 2024 by Chi Chim et al. [71] and investigated kdr mutations in the Nav gene, commonly associated with pyrethroid resistance, in populations from Yucatán. Immature stages of the mosquito were collected in Polaban, a rural area of the state, in 2021. Susceptibility bioassays were conducted using the CDC bottle bioassay and dose diagnostic, applying the recommended diagnostic dose for lambda-cyhalothrin (10 µg/bottle). Subsequent molecular assays were performed to detect target-site mutations. After 24 h of exposure, the mortality rate reached 100%, indicating that the population was susceptible to the pyrethroid, and 50 females, from those exposed to the insecticide, had their DNA extracted. Even though *Ae. albopictus* was susceptible to lambda-cyhalothrin, sequencing the corresponding Nav of transmembrane domains II–IV of the voltage-gated sodium channel revealed both synonymous and non-synonymous mutations were detected in the population: Thr1008 (ACG) and V1016A (GTA to GCA) in Domain II, and L1530S (TTA to TCA), I1410Thr (ATC to ACC), P1528S (CCU to TCU) and 1528S (TCC) in Domain III. No mutations were found in Domain IV. The lack of a susceptible lineage of *Ae. albopictus*, restricted the bioassays to a comparison of mortality between insecticide-treated bottles and ethanol-only controls, limiting the conclusions of this study.

#### 3.3.6. Panama

Carrera et al. (2024) [72] conducted a nationwide study in Panama, collecting immature stages of the mosquito in 16 urban and semi-urban areas, both in the indoor environment and the peridomestic area. Among those sites, *Ae. albopictus* was found in 13 localities: 24 Diciembre, San Isidro, El Coco, Sabanitas, Meteti, Aguadulce, Natá, Canto del Llano, San Félix, Las Tablas, Chitre, David and Puerto Armuelles. The Fraga colony, kept in the insectary of the IPK in Havana, Cuba, since 2012, was used as a susceptible reference lineage.

Insecticide resistance to the pyrethroids deltamethrin (0.03%), lambda-cyhalothrin (0.03%), cyfluthrin (0.15%) and permethrin (0.25%), the organochlorine DDT (4%), the organophosphates pirimiphos-methyl (0.25%), malathion (5%), chlorpyrifos-methyl (0.4%) and fenitrothion (1.0%) and the carbamate propoxur (0.1%) was assessed using the discriminant concentrations and exposure times of the WHO paper-impregnated tube test protocol from 2016. The populations of 24 Diciembre, San Isidro, El Coco, Sabanitas, Meteti, Canto del Llano, San Félix, Las Tabla and David showed possible resistance to deltamethrin, lambda-cyhalothrin, and cyfluthrin. Within these, Sabanitas, Canto del Llano, San Félix, Las Tablas and David also showed possible resistance to DDT, while Sabanitas presented possible resistance to permethrin as well as to DDT, suggesting potential cross-resistance mechanisms. For organophosphates, all populations showed possible resistance to the compounds tested, although confirmed resistance was observed only in the Canto del Llano population, specifically to pirimiphos-methyl and malathion. Regarding the carbamates, Sabanitas, San Félix and David showed possible resistance to propoxur.

In biochemical tests, enzyme activity was classified as unaltered (between 0 and 15%), incipient altered (between 15 and 50%), or altered (above 50%), over the reference lineages. Biochemical assays revealed altered activity levels of α-EST in the populations of San Isidro, Aguadulce, and Canto del Llano, whereas the populations of Meteti and Sabanitas showed incipient activity of the same enzyme. Altered β-esterase (β-EST) activity was recorded in Canto del Llano, supporting organophosphate resistance. Incipient altered AChE activity was observed exclusively in the Sabanitas population, whereas the populations of El Coco, Chitre and Sabanitas exhibited an incipient activity level of MFOs. No alterations in GST enzymatic activity were detected.

The elevated activity of esterases in the populations, especially α-EST, suggests their involvement in metabolic mechanisms that confer resistance to the tested insecticides. The incipient activity of MFOs may explain the possible resistance to pyrethroids observed. Based on the results of this and previous works with *Ae. aegypti*, the authors conclude that insecticide resistance appears to have increased in recent years across endemic regions. A key limitation of this study was the inability to perform molecular assays to detect any kdr mutations.

#### 3.3.7. Venezuela

Molina de Fernández et al. (2016) [73] conducted a study on *Ae. albopictus* populations collected during 2012, 2013 and 2014 in the immature stages (larvae and pupae) in two Venezuelan states, Araguara and Carabobo. Five colonies were established from these collections and subjected to biochemical and to the CDC bottle bioassays, following the Brogdon and McAllister methodology. The insecticides evaluated were DDT (200 μg/mL), malathion (100 μg/mL), fenitrothion (100 μg/mL) and lambda-cyhalothrin (6.25 μg/mL).

The authors recorded mortality every 15 min until 100% mortality was achieved, allowing them to determine the insecticide’s effect as a function of exposure time. All five populations were susceptible to DDT and lambda-cyhalothrin. Regarding malathion, one population exhibited full mortality (100%) within 30 min of exposure, while the remaining four required 45 to 60 min to reach the same outcome. For fenitrothion, two populations had 100% mortality within 30 min, and the other three had mortality from 45 to 60 min. These findings indicate the presence of incipient resistance to organophosphates in these populations.

To investigate the role of metabolic resistance mechanisms, synergist bioassays were conducted using piperonyl butoxide (PBO) in two of the more tolerant populations—one that reached full mortality in 45 min and another in 60 min. The addition of PBO reduced this time to 15 and 45 min, respectively, suggesting the involvement of oxidases in the detoxification process.

Finally, biochemical assays were performed to quantify the enzymatic activity associated with metabolic resistance. Statistically significant differences and higher activity were observed among the populations for all enzymes analysed (α- and β-EST, AChE, insensitive AChE and oxidases), further supporting the presence of metabolic resistance mechanisms in these populations.

## 4. Discussion

Insecticide resistance in *Ae. albopictus* has been extensively documented in temperate regions, where this species predominates and acts as the primary vector of arboviruses [76]. This has driven greater investment in research both within the species’ native habitat and in locations where it invaded [7,77,78,79,80,81,82,83,84,85,86]. In the Americas, however, only the United States, where the mosquito was first confirmed in 1983 [20], has this type of research well-documented [49].

The study conducted by Estep et al., [49] revealed that most populations were susceptible to pyrethroids such as permethrin, with only sporadic reports of low-intensity resistance. In contrast, reduced susceptibility or low-level resistance to organophosphates, especially malathion, has been more frequently detected, including historical resistance in Texas that persisted for many generations in the absence of insecticide pressure, suggesting minimal fitness costs [49,57,64].

Larval bioassays similarly revealed occasional resistance to chlorpyrifos and malathion, sometimes at higher levels than in adults. Several studies indicate that laboratory-detected resistance has not consistently translated into operational control failures and overall, the pattern across the country suggests that while *Ae. albopictus* in the U.S.A. can develop resistance to organophosphates, susceptibility to pyrethroids remains largely preserved [49,87].

Interpretation of insecticide resistance patterns in *Ae. albopictus* across Latin America and the Caribbean is limited by methodological heterogeneity, exposure metrics, diagnostic thresholds, and temporal gaps between studies, which limit direct comparisons across countries.

In Latin America and the Caribbean, our review revealed a heterogeneous and worrisome resistance landscape, characterized by limited studies and resistance patterns that may be influenced by regional insecticide application and cross-pressure from *Ae. aegypti* control strategies. Only seven of the 33 countries (21%) in the region have reported resistance data on this species, resulting in a geographically sparse and uneven evidence base. In addition, the heterogeneity in bioassay methodologies, such as larval *versus* adult bioassays, and bottle assays *versus* impregnated papers, limits direct comparisons across studies. Consequently, regional patterns discussed here are based on geographically sparse and skewed evidence and methodologically diverse data and should not be interpreted as representative of the entire region or as supporting robust pooled inferences.

Most reports came from Mexico (8) and Brazil (4), the first two countries that reported the presence of the vector in the region [21,22]. Single studies were found in Costa Rica, Cuba, Haiti, Panama, and Venezuela, where the mosquito was reported in the 2000s and 2010s [23,24]. This difference in the number of published papers indicates a geographic concentration of this type of research in countries where the species has been well reported for a longer period and where vector control programs have historically received greater attention [88].

The prevalence of the use of the organophosphate temephos directly correlates to its historical use in vector control programs in the region, indicating a possible bystander selection due to control programs focusing on *Ae. aegypti* [46,89,90]. This prolonged exposed pressure explains the reports of resistance found in Brazil by Prophiro et al. (2011) [58], 20 years after Wesson (1990) [57] reported full susceptibility to it. Notably, Prophiro et al. (2011) [58] used the *Ae. aegypti* Rockefeller strain as the susceptible reference when testing the larvae. Previous studies have already demonstrated that *Ae. albopictus* reference strains can be up to 2.5-fold more tolerant to insecticides than the Rockefeller strain [74]. Consequently, resistance ratios derived from heterospecific comparisons may systematically overestimate true resistance levels in the species, and, therefore, should be interpreted with caution.

This methodological limitation has important implications for interpreting resistance levels in *Ae. albopictus*. When the susceptible control strain is more sensitive to insecticides, the resulting resistance ratios may be overestimated, giving the impression of stronger resistance. Acknowledging this is essential, as it highlights the urgent need for a standardized susceptible *Ae. albopictus* strain and species-specific discriminating concentrations to improve the accuracy and comparability of resistance assessments.

A similar pattern was observed in Mexico, although the timeline appeared shorter. López-Solis et al. (2020) [66] reported temephos resistant populations merely a decade after Marina et al. (2010) [65] had confirmed the larvicide’s effectiveness against *Ae. albopictus*. This shift highlights how local operational practices and insecticide use histories differ across countries, influencing the speed and intensity with which resistance emerges.

In recent years, *Ae. albopictus’* presence has become frequent in urbanised areas, not only due to its high dispersal capacity but also because of its ability to adapt to new environments. Originally, it was primarily found in natural and peri-urban habitats, but it now coexists with *Ae. aegypti* in urban areas, where the selective pressure from chemical insecticides is more intense [5,6,13,14,15,16,17,91]. This may have contributed to the emergence of resistance, as observed with the exposure to temephos.

However, this does not appear to extend to biological larvicides, as Suter et al. (2017) [60] reported susceptible populations in Brazil to *Bti* and the combined product *VectoMax* CG^®^. Although Marina et al. (2010) [65] observed a reduced *Bti* efficacy in Mexico, this does not necessarily indicate resistance. Field evaluations are important for assessing the real effectiveness of a product, but laboratory bioassays are necessary to determine whether the reduction is due to resistance or external factors. In many cases, decreased efficacy may be related to the residual activity of the product in the field, which is influenced by environmental conditions such as temperature, sunlight exposure, and pH variations [92,93,94,95]. So far, no resistance to whole-crystal *Bti* has been detected in natural field populations of mosquitoes, including *Ae. aegypti* and *Ae. albopictus*, despite decades of operational use [27]. The presence of multiple toxins with distinct modes of action that act synergistically is regarded as the main factor preventing the emergence of resistance in most target insect species.

The status of resistance or susceptibility to adulticides has been more frequently documented than that of larvicides and shows a greater regional variation. Susceptibility to organophosphates was documented in Brazil and Mexico in early 90s [57,64], with similar findings in Haiti during the 2010s [63]. More recent studies in Mexico and Cuba have continued to show susceptibility [62,69].

López-Solís et al. (2020) [66] reported malathion resistance in *Ae. albopictus* from Tapachula, Mexico, a high-dengue-incidence city where malathion is widely applied in spatial spraying during dengue epidemics [96]. Resistance was also reported in Venezuela [73]. Both outcomes appear to result from selective pressure due to the insecticide use in *Ae. aegypti* control programs. In Venezuela, organophosphates, such as malathion and fenitrothion, have been the primary class for vector control since the 1970s [97], while in Tapachula, malathion has been routinely used since 2017 [98].

In addition to the pressure exerted by control programs, resistance selection may also be associated with the extensive use of pesticides in agricultural activities [99]. The intensive use of pesticides in Latin America and the Caribbean for both agricultural production and public health vector control has been documented and has resulted in widespread chronic exposure, particularly among populations living in agricultural communities [48]. Similar scenarios have been described on other continents as a key factor in cross-resistance in mosquitoes owing to constant exposure to compounds of the same classes used in public health [100,101]. Considering that these species can easily move between rural, peri-urban, and urban environments, the mobility of resistant alleles can be accelerated, increasing the risk of regional spread of resistance mechanisms and hindering control efforts.

In addition, current evidence suggests a growing number of reports documenting signs of pyrethroid resistance across the region. While initial studies from Mexico in the 1990s and from Haiti and Costa Rica during the 2010s documented full susceptibility [61,63,64], the situation has changed over the years. Multiple studies performed in Mexico between 2020 and 2024 detected low resistance levels [66,67,68,71] and similar results have been observed in Venezuela, Cuba and Panama in recent surveillance data [62,72,73].

Although Tancredi et al. (2020) [67] observed kdr mutations in Mexican populations, available molecular evidence across the regions remains limited and fragmented. Available evidence from the region indicates that kdr mutations in the Nav gene have been detected only sporadically. Surveys report no kdr alleles in populations from Costa Rica and Cuba, whereas Mexican populations have yielded substitutions at positions 410, 989 and 1534 (including F1534C), and Haitian populations exhibits high frequencies of V1011Met [61,62,63,67]. However, sampling remains patchy, many countries lack published assays (Venezuela, for example, is absent from the dataset because of methodological constraints), so geographic coverage is incomplete. Crucially, current data do not permit robust correlations between these Nav alleles and phenotypic insecticide resistance in Latin American and Caribbean *Ae. albopictus*; coordinated studies that pair molecular genotyping with standardized bioassays are therefore required to guide effective resistance-management strategies.

In contrast, metabolic resistance appears to be the most common mechanism in the studies, once biochemical assays from multiple countries have demonstrated altered enzyme activities associated with resistance [62,66,68,72,73]. It is important to highlight that these assays varied in methodology and sample size across studies. Once there are no standardized cut-offs, altered enzyme activity does not necessarily predict operational control failure. Consequently, this inference should be regarded as indicative rather than definitive.

The increased activity of MFOs has been well documented as a key contributor to pyrethroid resistance in *Ae. aegypti* [102,103,104,105], suggesting a similar, but less understood, association in *Ae. albopictus*. In addition, the increased activity of α- and β-EST, which are typically associated with organophosphate resistance in *Ae. aegypti* [106] seems to contribute to pyrethroid resistance in *Ae. albopictus* as well.

Few studies have been conducted with the carbamates propoxur and bendiocarb, and even fewer with the organochloride DDT against *Ae. albopictus* populations. Most available data available indicate susceptibility to both classes, with López-Solís et al. (2020) [66] and Contreras-Perera et al. (2021) [69] reporting susceptible populations to the carbamates in Mexico, Piedra et al. (2023) [62] reporting it in Cuba, and Molina de Fernández et al. (2016) [73] finding susceptible populations to DDT in Venezuela. In contrast, Carrera et al. (2024) [72] identified populations resistant to both propoxur and DDT in Panama.

The DDT resistance found in Panama [72] may be linked to the deltamethrin resistance observed in the same study, given that both insecticides share a common target-site mutation in the Nav gene [30]. Given that *Ae. albopictus* was first recorded in Panama in 2004, while DDT was used nationally until the early 2000s and pyrethroids were introduced soon after [24,107,108], direct selection by DDT on the species is unlikely. The observed resistance may have emerged as a result of the cross-resistance driven by the use of pyrethroids as already reported elsewhere [109].

The propoxur resistance reported in the same study [72] likely involves distinct mechanisms. A recent Chinese research on *Ae. albopictus* [110] suggested that resistance to carbamates is primarily metabolic, involving the overexpression or alteration in the genes of detoxifying enzymes, which is corroborated by the finding of increased activity of α-EST by Carrera et al. (2024) [72]. In *Ae. aegypti*, the G119S amino-acid substitution in the acetylcholinesterase (*ace-1*) gene conferring resistance to carbamates is extremely rare as it requires two concurrent mutations, and has so far been documented only once, in a population from India [111]. However, further genomic investigations are needed to determine the presence and distribution of *ace-1* variants in America and their contribution to resistance phenotypes in *Aedes* populations.

Finally, this review identifies three major limitations that currently hinder a comprehensive understanding of insecticide resistance in *Ae. albopictus* across this region. First, there is a marked scarcity of data from Latin America and the Caribbean, where research efforts have historically centred on *Ae. aegypti*, the primary arbovirus vector in this region. Second, most available studies do not include bioassays using newer insecticides (e.g., clothianidin, and flupyradifurone) or insecticide mixtures, which is a significant gap given their increasing relevance for vector control and the fact that classical test protocols are not recommended for several of these compounds. Third, and perhaps the most critical limitation, is the absence of a standardised susceptible reference strain for *Ae. albopictus*, equivalent to Rockefeller, Bora, or New Orleans for *Ae. aegypti*, which greatly restricts the comparability of susceptibility assays and resistance profiles across studies.

Thus, future surveillance efforts of insecticide resistance in *Ae. albopictus* should prioritize the use of standardized susceptible reference strains of the same species and/or internationally recognized diagnostic doses (e.g., WHO guidelines). In the absence of such strains and/or doses, studies should adopt interim strategies such as inter-laboratory calibration exercises and clearly report of mortality data, to ensure the comparability of results. Given the current scarcity of data, bioassays with commonly used insecticides (e.g., deltamethrin and malathion) should continue; however, this effort must be expanded to include newer insecticides and mixed formulations. These evaluations should be conducted through dose–response assays designed to establish species-specific diagnostic concentrations, using clearly described and harmonized protocols to guarantee the reproducibility across laboratories.

## 5. Conclusions

The reviewed literature indicates that several studies have reported a pattern of reduced susceptibility to insecticides in *Ae. albopictus* in Latin America and the Caribbean. However, direct comparisons over time and space remain challenging, as differences in bioassay methodologies, diagnostic doses, reference strains, and resistance metrics limit regional inferences. Across studies, biochemical assays frequently report elevated detoxification enzyme activity, suggesting that metabolic mechanisms are the predominant drivers of resistance in this region. Overall, these findings support the hypothesis that selective pressure from *Ae. aegypti* control efforts, which predominantly rely on the use of chemical compounds, may favour the selection of resistance alleles in *Ae. albopictus* in areas of sympatry. This could undermine the efficacy of current vector control strategies, especially given the increasing distribution of this vector in urban areas.

## Figures and Tables

**Table 1:** Interpretation criteria for biochemical and molecular resistance markers in *Aedes* mosquitoes.

Marker	Typical Metric Reported by Authors	Interpretation Commonly Adopted in the Literature	Ref.
MFOs (e.g., P450s)	Activity or gene expression significantly higher than susceptible reference population	Role in metabolic resistance, mainly to pyrethroids, organophosphates and carbamates	[51,52,53]
α-EST/β-EST	Activity or gene expression significantly higher than susceptible reference population	Role in metabolic resistance, mainly to pyrethroids, organophosphates and carbamates	[51,52,53]
GST	Activity or gene expression significantly higher than susceptible reference population	Role in metabolic resistance, mainly to pyrethroids, organophosphates and carbamates	[51,52,53]
Ace	Reduced acetylcholinesterase inhibition	Role in target-site insensitivity to organophosphates and Carbamates	[51,53,54]
Nav mutations (kdr)	Presence of non-synonymous mutations in the canal sodium channel	Role in target-site resistance to pyrethroids and organochlorides	[51,53,54]

**Table 2:** Summary of bioassays conducted on *Aedes albopictus* populations across Latin America and the Caribbean nations for insecticide resistance assessment.

Country	Field Populations	Insecticide	Type of Assay	Outcome	Year	Ref.
Brazil	Five collected in the states of Espírito Santo and São Paulo	Temephos, Chlorpyrifos, Fenitrothion, Fenthion, Malathion	Larval bioassay ^1^*	Only one population showed resistance to chlorpyrifos, while the remaining were susceptible to the other insecticides evaluated	1990	[57]
	From the municipalities of Ubiratã, Santa Helena, Foz do Iguaçu and Tubarão	Temephos	Larval bioassay ^2^*	Incipient resistance detected for all insecticides tested	2011	[58]
	One collected in Rio de Janeiro	Triflumuron	Larval bioassay ^1+^	No resistance detected	2013	[59]
	From Sítio dos Pintos, Recife	Bti, Cry11Aa, Cry4Ba; Lysinibacillus sphaericus; VectoMax CG^®^	Larval bioassay ^2^ˣ	No resistance detected	2017	[60]
Costa Rica	From Sarapiquí	-	Molecular assay	No kdr mutation was found	2015	[61]
Cuba	Plaza and Boyeros, collected in Havana	Bendiocarb, Propoxur; Cypermethrin, Deltamethrin, Lambda-cyhalothrin; Chlorpyrifos, Temephos	CDC bottle bioassay, larval bioassay and biochemical assay ^2^*	Both populations showed high resistance to temephos and incipient resistance to deltamethrin. Plaza population also exhibited resistance to lambda-cyhalothrin. All populations remained susceptible to the other insecticides. Increased α-EST and GST activity was detected	2023	[62]
Haiti	From two sites of Port-au-Prince	Permethrin	CDC bottle bioassay, molecular assay and biochemical assay ^1#^	No resistance was detected; however, elevated levels of oxidases, α-esterases, GST, and AChE were observed. Molecular assays detected no mutations.	2012	[63]
Mexico	Collected from three sites of Matamoros and one site from Reynosa	Resmethrin, Permethrin; Chlorpyrifos, Malathion	Bottle bioassay ^2§^	No resistance detected	1996	[64]
	Tested the efficacy of the products during the rainy and the dry season	Spinosad, Bti; Temephos	Larval bioassay ^1^	Spinosad and temephos were effective in preventing Ae. albopictus development in both seasons, whereas the Bti suspension showed reduced activity over time	2010	[65]
	Collected from two urban sites in Tapachula	Bendiocarb, Propoxur; Permethrin, Deltamethrin; Chlorpyrifos, Malathion, Temephos	CDC bottle bioassay, larval bioassay and biochemical assay ^2#^*	Both populations were susceptible to all evaluated CAs, showed incipient resistance to PYs and chlorpyrifos, and resistance to malathion and temephos. Elevated EST, monooxygenase, and AChE activity was detected at both sites	2020	[66]
	182 mosquitoes collected in Chiapas	-	Molecular assay	Kdr mutations were detected at positions 410, 989 and 1534	2020	[67]
	Eleven collected in Tapachula and in rural towns	Permethrin	CDC bottle bioassay, molecular assay and biochemical assay ^2+^	All populations showed a low level of resistance, and all tested mosquitoes were homozygous for the wild-type F1534 allele	2021	[68]
	From an area with a confirmed presence of Ae. albopictus	Bendiocarb, Propoxur; Chlorpyrifos, Malathion; Permethrin, Deltamethrin	CDC bottle bioassay ^2#^	No resistance detected	2021	[69]
	Cancún, Chetumal, Playa del Carmen and José María Morelos from Quintana Roo	Temephos	Larval bioassay ^1#^	The populations from Cancún and José María Morelos showed tolerance to the larvicide, whereas the remaining two were susceptible	2022	[70]
	Collected in Polaban, Yucatan, Mexico	Lambda-cyhalothrin	CDC bottle bioassay and molecular assay ^1#^	Despite susceptibility to PYs, both synonymous and non-synonymous mutations were detected in the population	2024	[71]
Panama	Thirteen collected across the country	Propoxur; Deltamethrin, Cyfluthrin, Lambda-cyhalothrin, Permethrin; DDT; Pirimiphos-methyl, Malathion	Impregnated paper bioassay and biochemical assay ^2#^	Possible resistance to PYs, DDT, OPs, and propoxur was detected in several populations, with confirmed resistance to pirimiphos-methyl and malathion in one population, accompanied by elevated EST activity.	2024	[72]
Venezuela	Santos Michelena, Carabobo, Capital District, Zamora and Mario Briceno Iragorry collected from Araguara and Carabobo	Lambda-cyhalothrin; DDT; Fenitrothion, Malathion	CDC Bottle bioassay and biochemical assay ^1^ˣ^^^	All populations were susceptible to DDT and lambda-cyhalothrin. Incipient resistance to OPs was detected in MBI and DC, whereas the remaining three were susceptible. Elevated EST activity was observed	2016	[73]

Legend: AChE = Acetylcholinesterase, CA = carbamate, EST = Esterase, GST = Glutathione S-transferase, OP = organophosphate, PY = pyrethroid. ^1^ No susceptible reference strain used. ^2^ Susceptible reference strain included. * Diagnostic dose determined by the laboratory. **^+^** Dose–response assays performed. ^x^ Insufficient information on dose determination. ^#^ Diagnostic doses based on WHO/CDC guidelines. ^§^ Doses determined using the Plapp vial technique. ^^^ Synergists were used to further investigate resistance mechanisms.

## Data Availability

No new data were created or analyzed in this study. Data sharing is not applicable to this article.

## Supplementary Materials

The following supporting information can be downloaded at: https://www.mdpi.com/article/10.3390/insects17030264/s1, Table S1: Methodological characteristics and main outcomes of bioassays conducted on *Aedes albopictus* populations across Latin America and the Caribbean nations for insecticide resistance assessment.
